# Molecular Evolution of the Glycosyltransferase 6 Gene Family in Primates

**DOI:** 10.1155/2016/9051727

**Published:** 2016-12-04

**Authors:** Eliane Evanovich, Patricia Jeanne de Souza Mendonça-Mattos, Maria Lúcia Harada

**Affiliations:** ^1^Laboratório de Genética Humana e Médica, Instituto de Ciências Biológicas, Universidade Federal do Pará, Belém, PA, Brazil; ^2^Laboratório de Biologia Molecular “Francisco Mauro Salzano”, Instituto de Ciências Biológicas, Universidade Federal do Pará, Belém, PA, Brazil

## Abstract

Glycosyltransferase 6 gene family includes ABO, Ggta1, iGb3S, and GBGT1 genes and by three putative genes restricted to mammals, GT6m6, GTm6, and GT6m7, only the latter is found in primates. GT6 genes may encode functional and nonfunctional proteins. Ggta1 and GBGT1 genes, for instance, are pseudogenes in catarrhine primates, while iGb3S gene is only inactive in human, bonobo, and chimpanzee. Even inactivated, these genes tend to be conversed in primates. As some of the GT6 genes are related to the susceptibility or resistance to parasites, we investigated (i) the selective pressure on the GT6 paralogs genes in primates; (ii) the basis of the conservation of iGb3S in human, chimpanzee, and bonobo; and (iii) the functional potential of the GBGT1 and GT6m7 in catarrhines. We observed that the purifying selection is prevalent and these genes have a low diversity, though ABO and Ggta1 genes have some sites under positive selection. GT6m7, a putative gene associated with aggressive periodontitis, may have regulatory function, but experimental studies are needed to assess its function. The evolutionary conservation of iGb3S in humans, chimpanzee, and bonobo seems to be the result of proximity to genes with important biological functions.

## 1. Introduction

Glycosyltransferases catalyze the biosynthesis of glycoconjugates and polysaccharides by addition of sugar residues to an acceptor substrate that produce important antigens in the signaling process and recognition by the immune system [1], consisting of more than 90 carbohydrate-active enzymes, grouped by protein similarities in the CAZY database (http://www.cazy.org/GlycosylTransferases.html). Glycosyltransferase 6 are type II transmembrane proteins localized in the Golgi complex. They have a general structure with a cytoplasmic tail, a transmembrane domain, and a large catalytic domain [1–3]. The family includes functional and nonfunctional proteins encoded by ABO, Ggta1, iGb3S, and GBGT1 genes and by three putative genes restricted to mammals: GT6m6, GTm6, and GT6m7; only the latter is found in primates [3].

ABO gene encodes A and B transferases that add N-acetyl-D-galactosamine (GalNAc) or D- galactose (Gal) to H substance that produce A or B antigens, respectively. O allele is nonfunctional [4, 5]. Individuals with the O phenotype exhibit more resistance to severe malaria caused by* Plasmodium falciparum* than others with A and/or B types [6–8]. Nonetheless, analysis of this locus in primates showed that the alleles were maintained by balanced selection [9].

*Ggta1* gene encodes the enzyme *α*1,3-galactosyltransferase (*α*1,3-GT). It transfers UDP-Gal to N-acetyllactosamine and produces *α*-Gal epitope. Catarrhini (human, apes, and Old World monkeys) produce anti-Gal antibody [10, 11]. According to Galili [12] the inactivation of that gene assured the emergence of this lineage. However, the functionality of Ggta1 is maintained in noncatarrhine mammals [13]. The synthesis of *α*-Gal epitopes was also attributed to* iGb3S* gene in mouse. However, this gene encodes isogloboside b3 synthase by adding UDP-Gal to lactosylceramide that produces isogloboside b3 [14].

*GBGT1 *gene encodes the Forssman synthase (FS) via GalNAc addition to globotriaosylceramide. The result of this reaction is Forssman antigen, absent in catarrhine primates due two nonsynonymous substitutions at residues 230 (G > S) and 269 (Q > R) [5, 15].

GT6 member putative genes do not appear to produce an enzyme with catalytic activity due to lack of six motifs LBR-B, LBR-C, LBR-F, LBR-G, LBR-H, and LBR [3].

Casals et al. [16] analyzed the molecular evolution of GBGT1, iGb3S, and GTm7 in two human populations and surprisingly described that these supposed pseudogenes evolve under positive selection. Disagreeing with what would be expected for a nonfunctional sequence, although initially described as pseudogenes, some studies have shown the possibility of GT6m7 being functional. Schaefer et al. [17] found an association between this pseudogene and aggressive periodontitis in three European populations. The same association was strengthened by Hashim et al. [18].

According to Svensson et al. [19], three criteria are important to establish a pseudogene: (1) detrimental mutation (mutations, stop codon, frameshift, splice-site alterations, etc.); (2) number of nonsynonymous substitutions per nonsynonymous site (Ka/Ks ratio) that indicate absence of selective pressure; and (3) when the sequences are not overlapping any known gene.

Nevertheless, there are some controversial facts surrounding these criteria: some discoveries have found that some putative pseudogenes participate in DNA, RNA, and protein regulation and that their gene sequences were conserved for millions of years, when they should be evolving neutrally. Therefore, reviewing the pseudogene condition seemed to be important.

Like some of the GT6 genes are related to the susceptibility or resistance to various parasites, their evolutionary and functional analysis is important. Thus, we decided to investigate (i) the selective pressure on the GT6 paralogs genes in primates; (ii) the cause of the conservation of iGb3S pseudogenes in human, chimpanzee, and bonobo; and (iii) the functional potential of the* GBGT1 *and* GT6m7 *in catarrhines based on the literature and annotated protein database.

## 2. Material and Methods

### 2.1. Database and Alignment

The sequences used were retrieved from NCBI (https://www.ncbi.nlm.nih.gov/) and/or Ensembl databases (http://www.ensembl.org/index.html). The identification of the species used for each database and their accession numbers are presented in Additional file (Table S1 in Supplementary Material available online at http://dx.doi.org/10.1155/2016/9051727).

We analyzed only exons 6 and 7 of the ABO gene which encode the catalytic domain of the protein (comprising 823 of 1062 bp, resp.). Exons 1 to 5 are small (their all length is 237 bp) and they are absent or removed by alternative splicing of transcripts in many different tissues of mammals. The sequences were aligned using the MUSCLE of the MEGA 6 [20]. The best-fit model of evolution for each gene was estimated by Modeltest 3.7 [21] and the phylogenetic tree for 100 replicates was built using PhyML 3.0 [22]. Nucleotide diversity (*π*) was calculated by MEGA 6 [20].

### 2.2. Detection of Recombination Breakpoints

Recombination was performed by GARD methods available at the http://www.datamonkey.org/ server [23] under HKY85 model using the Kishino-Hasegawa test (KH). The rate variation was implemented by a general discrete distribution with three rate classes [23].

### 2.3. Analysis of Molecular Evolution

Molecular evolution was verified by Codeml program implemented in PAML 4 package [24] and by HyPhy in Datamonkey server [23]. Codeml was initially running the M0 model to obtain phylogenetic trees which subsequently were inputted in site models and “free-ratio” branch model [25, 26].

Likelihood ratio test (LRT) with *χ* ^2^ (*α* = 0.05) was used to compare null models with the alternative models (M1a versus M2a and M7 versus M8) and M0 versus free-ratio model [25–28] and LRT with critical values of 2.71 at 5% and 5.41 at 1% for comparing the nested pair M8a versus M8 test. SLAC, FEL, and FUBAR methods of the Hyphy were used to identify sites under positive or purifying selection. Evidence of episodic positive selection was verified by MEME (applied to individual sites) and BUSTED (applied to gene-wide with primates as foreground branch) [29].

These methods use different confidence index as *p* value, Bayes factor, and posterior probability. *p* value was significant if it is less than or equal to 0.05 (BUSTED, SLAC, FEL, and MEME models), Bayer factor ≥ 50 (REL), and posterior probability ≥ 95% (FUBAR). The models of evolution used in these analyses were REV (for* ABO*,* Ggta1*, and* GT6m7*) and HKY85 (for* GBGT1* and* iGb3S*).

### 2.4. Annotated Protein Database

To obtain more functional information, tissue expression, and protein interactions on each of the genes, we searched databases neXtProt (https://www.nextprot.org/), UniProtKB (http://www.uniprot.org/), GeneCards (http://www.genecards.org/), and String (http://string-db.org/).

## 3. Results and Discussions

GARD method found evidence of recombination only in the* GT6m7* sequence at position 190. PARRIS algorithm indicates that the genes are strong purifying selection, and BUSTED does not find evidence of diversifying selection under each whole gene. However, some sites were identified by the MEME as under episodic selection pressures (Table 1). The results obtained for each of the genes are shown in Table 1.

### 3.1. ABO

ABO analyses performed by Codeml models inferred the site 266 under positive selection. This codon position is crucial for differentiating A and B alleles in primates [9, 30]. Although the M8 is not concise as the M2a model [23], the presence of diversifying and positive selection was indicated by MEME and “free-ratio” model. MEME algorithm suggested diversifying selection on site 268 (*p* value of 0.002). “Free-ratio” test showed that some branches evolve on positive selection. The results obtained corroborate to balancing selection described by Ségurel et al. [9]. According to some authors, certain alleles may act against pathogens and are maintained during the evolution [31–34]. In human populations, individuals with A or B types seem to be less susceptible to infections caused by* Helicobacter pylori *and* Vibrio cholerae *compared to the carriers of O allele [32, 35]; in contrast, greater resistance to severe malaria caused by* Plasmodium falciparum* in blood group O individuals due to reduced rosette formation in erythrocytes was observed [6, 36]. Results are shown in additional file: Tables S2–S4 and Figure S1.

### 3.2. Ggta1

None of the sites found by algorithms are involved in the protein activity in accordance with the inferred structure by Gastinel et al. [37] for bovine 1,3-Gal. Positive selection was indicated by comparison of M7 versus M8 models (LTR = 6.367; *p* < 0.05) in position 109 (E > K); however, it substitution is restricted to Gtta1 pseudogene of Angola colobus, thus expected.

250 and 351 site codons were found on pervasive diversifying selection by MEME and, respectively, by SLAC and FEL. Codon 250 is localized in *α*1,3-GalT catalytic pocket. It is involved in the stability of the C-terminal segment [37] and is a binding site for disaccharide acceptor substrates [38]. The squirrel monkey *α*1,3-GalT presents substitution of tryptophan to phenylalanine in this residue, but this change does not seem to have a large impact on the catalytic activity of the enzyme [38]. Codon 351 is near to C-terminal portion and in this analysis it presents very polymorphism, having polar and nonpolar amino acids in different mammals, but they do not seem to result in major changes in protein structure.

Results of “free-ratio” test indicate different selective pressures between the several lineages, especially in howler that shows the highest *ω* values (0.64), similar to results found by Koike et al. [13] (additional file: Tables S5–S7 and Figure S2).

### 3.3. iGb3S

**Figure 1:**
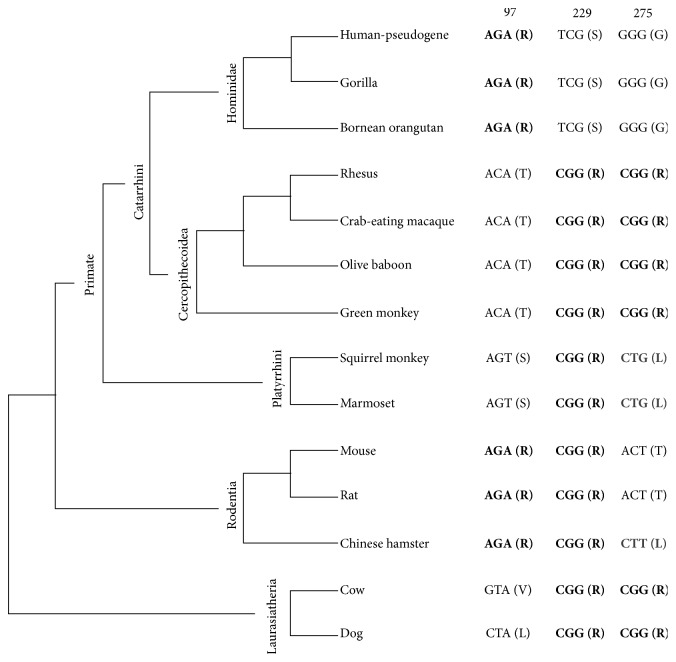
Phylogenetic tree for* iGb3* gene indicated three sites under diversifying selection in accordance with MEME. The sites that are shared by different taxa are highlighted in bold. Tree constructed using PhyML, 100 replicates, under GTR + G model.* Lnl *= −4263.35. Values of branches sizes and bootstrap were omitted.

Only the site 97 was pointed out as being under positive selection by FEL. It was also indicated by the MEME, such as sites 136, 229, and 275. Among them, 97, 229, and 275 positions are most relevant information, because amino acids are preserved in different taxa showing a strong selective pressure on protein in mammals (Figure 1) [39]. Substitutions in these residues are not involved in important enzymatic processes. However, the polarity changes involving residue 275 as observed in iGb3S protein of the Old World monkeys (G275R) may result in some change in conformational entropy of the backbone in the local coil of the protein (see [40]), because this residue is in a hydrophobic site on *α*-helix (*α*5).

This gene is inactive in humans due to multiple mutations, including Y252N and L187P substitutions, but the high conservation of the putative pseudogene in human populations (nucleotide diversity,*π* = 0.0010) was reported by Casals et al. [16].

*iGb3S* has no evidence of recombination and it is syntenically conserved in mammals. Moreover, it is flanked by two conserved genes (ZNF362, Zinc finger protein 362 and PHC2, polyhomeotic homolog 2 genes), suggesting that their conservation in humans may result from their chromosomic position that is under strong selective pressure (additional file: Tables S8–S10).

### 3.4. GBGT1

PARRIS indicates 82% sites under purifying selection (*ω* mean = 0.270) and particularly in Catarrhini that present low diversity (*π= *0.030). (Results are shown in additional file: Table S11–S13.) These primates are Forssman-negative, instead of Forssman glycolipid. Some cells of these primates show the precursor glycolipids globotriaosylceramide and globoside that are used as a binding site for bacteria, viruses, and toxins [15, 41–44]. This is apparently an adaptive disadvantage, but it can be offset.

For instance, individuals whose expression is Forssman antigen (ABO subgroup A_pae_) [19] present protection against Shiga toxin 1 (Stx1) and vulnerability to Stx2a toxin, variant of Stx2 though [45, 46]. Louise and Obrig [47] analyze patients with hemolytic uremic syndrome (HUS) caused by* Escherichia coli* O157 : H7 and observed that Stx2a toxin is 1000 times more cytotoxic than Stx1. Other epidemiological data using baboon and mouse models also noticed it [47–49]. It is possible that it is an evolutionary advantage, presenting a key role in the evolution of Catarrhini.

### 3.5. GT6m7

Our results indicated high conservation of nucleotides and purifying selection (*ω* mean of 0.475) as found by Casals et al. [16], although positive selection was detected at codon 126 by SLAC, FEL, MEME, and FUBAR (additional file: Tables S14–S16).

There is missing information on the protein structure, although its higher expression in the testis, leukocytes, and gingiva [17] is quite instigating. The most relevant information obtained was related to its regulatory potential, which is explained below.

In a genome-wide association study (GWAS), Schaefer et al. [17] found relation between an allele of SNP rs1537415 (localized at intron 2 of* GT6m7*) to aggressive periodontitis (AgP) in three European populations. More recently, this association was found in Sudanese population [18]. Schaefer et al. [17] using computational analysis reported that substitution from C to G in SNP rs1537415 may decrease the affinity between the transcriptional factor GATA-3 and its binding site.

We submitted intron 2 to BLASTN Ensembl against Human GRCh38 and we found* a *fragment of 353 bp (121 bp upstream to SNP rs1537415) with greater identity than 90% to the following regulatory sequences: ADGRL3-AS1 (adhesion G protein-coupled receptor L3 antisense RNA), RUNX1 (runt-related transcription factor 1), SRBD1 (S1 RNA binding domain 1 gene), CTND2 (catenin delta 2), and FEZ2 (Fasciculation and Elongation Protein Zeta 2). The values obtained for the five best hits are in additional file: Figure S3.

In recent decades, the function of some mammalian pseudogenes was clarified, acting at different levels, from DNA to protein [50–55]. For instance, the transcription of the* Makorin1* gene is regulated by its pseudogene paralogous* Makarin1-p1* [55]*; *and HMGA1 (high mobility group A1) presents regulatory action on the insulin receptor gene (INSR) [55]. The data presented suggest that GT6m7 also has some functionality, but experimental data are needed.

## 4. Conclusion

As shown in this paper, the purifying selection is prevalent in primate GT6 genes and has a low diversity (*ω* mean = 0.342 and *π* mean = 0.0822). Surprisingly, their retention was found even in taxa where one of the genes is inactivated. ABO,* Ggta1*, and* GBGT1* genes appear to be under strong selective pressures exerted by pathogens. ABO* locus* evolved in a balanced manner in primates with the maintenance of the three major alleles in at least ten species, including human, orangutan, and squirrel monkey.* Ggta*1 gene also gives advantage against pathogens with expression of *α*-Gal epitopes or anti-Gal antibody [12]. Similarly, the absence of expression of Forssman antigen is relevant against bacterial infections. However, GT6m7 and* iGb3S* seem to evolve differently. GT6m7, in primates, may have regulatory function. Otherwise, the maintenance of* iGb3S* in humans, chimpanzee, and bonobo seems to be the only result of the proximity to genes with important biological functions.

## Supplementary Material

Supplementary materials include informations about used samples, such as all table and figures of the results.‏

## Figures and Tables

**Table 1:** Codons under positive selection or diversifying selection with values significant obtained by Codeml or HyPhy methods.

Gene	Codon position	Method
*ABO*	266	M7-M8
	268	MEME
*Ggta1*	109	M7-M8
	250	MEME, SLAC
	361	FEL, MEME
*iGb3S*	96	FEL, MEME
	135, 228, 233	MEME
*GBGT1*	18, 21, 237, 266	MEME
*GT6m7*	12	FEL, MEME
	96	SLAC, FEL, MEME, FUBAR
