# Supplementary material for: Molecular Evolution of the Glycosyltransferase 6 Gene Family in Primates
Source: Biochem Res Int. 2016 Dec 4;2016:9051727. doi: 10.1155/2016/9051727 (PMC5164903; doi:10.1155/2016/9051727)
Supplement: Supplementary file 1 — Supplementary materials include informations about used samples, such as all table and figures of the results.‏ [file 9051727.f1.pdf]

Additional file

Table S1 - Samples, theirs genbank/ Ensembl access numbers and phenotype

	ABO	
**Accession number NCBI/ Ensembl**	**Species**	**Phenotype**
	**PRIMATE**	
	CERCOPITHECIDAE	
AH008342	*Macaca fascicularis*	A
XM_005580531	*Macaca fascicularis*	B
AH0082202	*Macaca fascicularis*	O1
NW_001101647	*Macaca mulatta*	B
XM_003918629	*Papio anubis*	A
AH0066972	*Papio anubis*	B
	HOMINIDAE	
ENSGGOT00000032150	*Gorilla gorilla*	B
NM_020469.2	*Homo sapiens*	A1
JN791441.1	*Homo sapiens*	B
AF021846	*Pan troglodytes*	O
ENSPTRT00000039844	*Pan troglodytes*	
NM_001194925	*Pan troglodytes*	A
	PLATYRRHINI	
XM_003732072	*Callithrix jacchus*	
XM_003732072	*Callithrix jacchus*	
XM_003732072	*Aotus nancymaae*	
	STREPSIRRHINI (PROSIMIANS)	
NW_007079802	*Tarsius syrichta*	
ENSOGAT00000026266	*Otolemur_garnettii*	
	RODENTIA	
NM_0307184	*Mus musculus*	
AF469946	*Rattus norvegicus*	A
	LAGOMORPHA	
XM_ 002721538	*Oryctolagus cuniculus*	

	CARNIVORA	
XM_ 5483863	*Canis lupus familiaris*	ABO1
	CETARTIDACTYLA	
NM_001077926	*Bos taurus*	
	GBGT1	
**Accession number NCBI/ Ensembl**	**Species**	**Phenotype**
	**PRIMATE**	
	CERCOPITHECIDAE	
ENSCSAT00000013238	*Chlorocebus sabaeus*	
ENSMMUT00000021419	*Macaca mulatta*	
ENSPANG00000018174	*Papio anubis*	
	HOMINIDAE	
XM_004048799	*Gorilla gorilla*	Variant 1
ENSGGOT00000003467	*Gorilla gorilla*	
NM_021996	*Homo sapiens*	Variant 1
AY358175.1	*Homo sapiens*	
ENSPTRT00000039837	*Pan troglodytes*	
ENSPPYT00000023030	*Pongo abelii*	
	PLATYRRHINI	
XM_009004770.1	*Callithrix jacchus*	Variant X1
XM_010346403.1	*Saimiri boliviensis boliviensis*	Variant X1
	STREPSIRRHINI (PROSIMIANS)	
NW_007079802.1	*Tarsius syrichta*	
	RODENTIA	
NM 139197	*Mus musculus*	
ENSCPOT00000004927	*Cavia porcellus*	
	CARNIVORA	
XM_014116859.1	*Canis lupus familiaris*	
	CETARTIDACTYLA	
ENSOART00000005732	*Ovis aries*	
	GGTA1	
**Accession number**	**Species**	**Phenotype**

**NCBI/ Ensembl**		
	**PRIMATE**	
	CERCOPITHECIDAE	
XM_011927427.1	*Colobus angolensis palliatus*	like
	HOMINIDAE	
XM_003777440.2	*Pongo abelii*	like
	PLATYRRHINI	
DQ985356.1	*Alouatta caraya*	
AF384428.1	*Callithrix jacchus*	
AY034181.1	*Sapajus apella*	
XM_010335883	*Saimiri boliviensis boliviensis*	Variant X1
	STREPSIRRHINI (PROSIMIANS)	
AY126667	*Lemur catta*	
DQ985357	*Loris tardigradus*	
ENSOGAT00000001894	*Otolemur garnettii*	
ENSMICT00000016106	*Microcebus_murinus*	
XM_008049783	*Tarsius syrichta*	
	RODENTIA	
ENSSTOT00000005449	*Ictidomys_tridecemlineatus*	
AF297615	*Mus musculus*	
AF488784	*Rattus norvegicus*	
	CARNIVORA	
XM_014116859.1	*Canis lupus familiaris*	
NP_001009308.1	*Felis catus*	
	LAGOMORPHA	
XM_002720464	*Oryctolagus cuniculus*	
	CETARTIDACTYLA	
XM_010810167	*Bos taurus*	
AY447039	*Ovis aries*	
L36535	*Sus scrofa*	

iGb3S

Accession number NCBI/ Ensembl	Species	Phenotype

	**PRIMATE**	
	CERCOPITHECIDAE	
ENSCSAT00000017461	*Chlorocebus sabaeus*	
XM_005544050	*Macaca fascicularis*	
ENSMMUT00000004622	*Macaca mulatta*	
XM_003891544	*Papio anubis*	
	HOMINIDAE	
ENST00000442999	*Homo sapiens*	
ENSGGOT00000003612	*Gorilla gorilla*	
ENSPPYT00000001874	*Pongo abelii*	
	PLATYRRHINI	
ENSCJAT00000002165	*Callithrix jacchus*	
XM_003937545.1	*Saimiri boliviensis boliviensis*	
	RODENTIA	
XM_003500624	*Cricetulus griseus*	
ENSMUST00000030585	*Mus musculus*	
ENSRNOT00000007851	*Rattus norvegicus*	
	CARNIVORA	
XM_544424	*Canis lupus familiaris*	
	CETARTIDACTYLA	
XM_005203048	*Bos taurus*	

GT6m7

**Accession number NCBI/ Ensembl**	**Species**	**Phenotype**
	**PRIMATE**	
	CERCOPITHECIDAE	
NM_001283893	*Macaca fascicularis*	
ENSNLET00000011300	*Nomascus leucogenys*	
XM_008005684	*Chlorocebus sabaeus*	Variant_X1
XM_009187871	*Papio anubis*	
XM_010372750	*Rhinopithecus roxellana*	
	HOMINIDAE	
NM_182974	*Homo sapiens*	

XM_002820374	*Pongo abelii*	
ENSGGOT00000007894	*Gorilla gorilla*	
XM_008970052	*Pan paniscus*	
XM_528458	*Pan troglodytes*	
	PLATYRRHINI	
XM_009005825	*Callithrix jacchus*	
XM_003941373	*Saimiri boliviensis boliviensis*	
	STREPSIRRHINI (PROSIMIANS)	
XM_012782089.1	*Microcebus_murinus*	Variant X1
ENSOGAG00000030414	*Otolemur garnettii*	
XM_008070666.1	Tarsius syrichta	
XM_012654726.1	*Propithecus coquereli*	
	RODENTIA	
NM_001039095	*Mus musculus*	
NM_001106559	*Rattus norvegicus*	
	CARNIVORA	
XM_548383	*Canis lupus familiaris*	
	CETARTIDACTYLA	
NM_001075928	*Bos taurus*	
ENSSSCG00000005758	*Sus scrofa*	

Table S2 - Tests of positive selection codons in the *ABO* protein by pairs M0-M1a, M1a-M2a, M7-M8 and M8-M8a (PAML).

Model	lnL[a]	df[b]	LRT[c]	Estimates of Parameters[d,e]	Selected Sites	ω and SE
M0	-4046.235	1	213.87**	$\kappa= 3.591$, $\omega= 0.101$	Not Allowed	
M1a	-3939.301			$\kappa= 4.030$, $p_0= 0.825$, $p_1= 0.174$, $\omega_0= 0.048$, $\omega_1= 1.000$	Not Allowed	
		2	1.67			
M2a	-3938.466			$\kappa= 4.027$, $p_0= 0.824$, $p_1= 0.170$, $p_2= 0.005$, $\omega_0= 0.048$, $\omega_1= 1.000$, $\omega_2= 4.053$	None	
M7	-3909.200			$\kappa= 3.821$, $p= 0.226$, q= 1.409	Not Allowed	
		2	11.97**			
M8	-3903.215			$\kappa= 3.828$, $p0= 0.971$, ($p1= 0.029$), $p= 0.290$, $q= 2.531$, $\omega= 1.732$	266	1.717 +- 0.543
			3.61*			
M8a	-3905.020			$\kappa= 3.821$, $p0= 0.956$, ($p1= 0.044$), $p= 0.296$, $q= 2.902$, $\omega= 1.000$	None	

[a] The likelihood indicates the fit of the data to the different models; [b] Degree of freedom for $\chi 2$ test; [c] Result of the Likelihood ratio test amont the pair models M1a-M2a, M7-M8 and M8-M8a. *Indicate statistically significant at 95% level and **indicate statistically significant at 99% level ;[d] Pi denotes the proportion of sites falling in sites class ω_1; [e] Parameters *p* and q are the shape parameters of the beta distribution which underlies M7, M8 and M8a models

Table S3 - Results obtained by PARRIS for *ABO* protein (LRT = 0.00015)

Null model (M1)			
Rate Class	1	2	Summary
ω	0.05	1.00	Mean : 0.237
Prob.	0.805	0.195	Std.Dev : 0.375

Alternative model (M2)				
Rate Class	1	2	3	Summary
ω	0.05	1.00	4.41	Mean : 0.238
Prob.	0.805	0.195	0.000	Std.Dev : 0.375

Table S4- Codons under positive selection obtained by Hyphy algorithms for *ABO* protein (values significant statistical are in highlighted in bold).

**Codon**	**SLAC dN-dS**	**SLAC p-value**	**FEL dN-dS**	**FEL p-value**	**MEME ω^{+}**	**MEME p-value**	**FUBAR dN-dS**	**FUBAR Post. Pr.**
130	-0.135	0.677	-0.276	0.723	>100	**0.008**	-0.102	0.312
268	0.738	0.571	-0.283	0.776	>100	**0.002**	-0.126	0.395

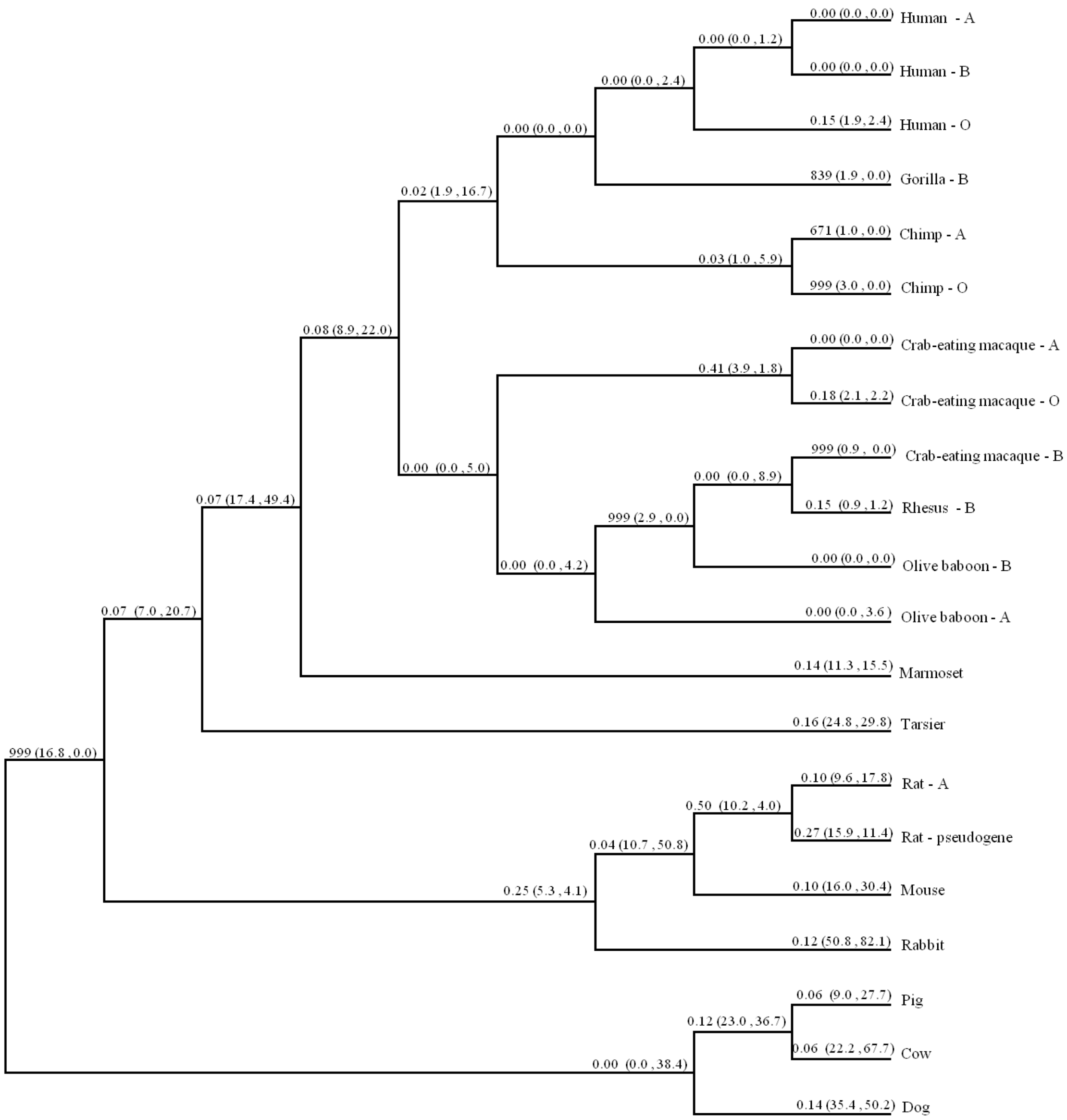

Figure S1 - Estimative of the Free-ratio model for the ABO protein. Samples with known phenotypes are indicated. The model estimated 594.1 and 116.9 nonsynonymous and synonymous sites, respectively. (M0 vs.Free-Ratio: *lnL*=- -4046.235288 and 4014.934, respectively; LRT = 62.6036; df= 39; $0.01 < p < 0.05$). The values on each branches corresponding to ω, nonsynonymous and synonymous sites, respectively. ω values < 0.5 indicate purifying selection, ω values > 0.5 is relaxed purifying selection and ω values > 1.0 is on positive selection.

Table S5- Tests of positive selection codons in the *Ggta1* protein by pairs M0-M1a, M1a-M2a, M7-M8 and M8-M8a (PAML).

Model	lnL[a]	df[b]	LRT[c]	Estimates of Parameters[d,e]	Selected Sites	ω and SE
M0	-5363.619	1	116.52**	κ= 4.182, ω= 0.264	Not Allowed	
M1a	-5305.348			κ= 4.417, p_0= 0.797, p_1= 0.202, ω_0= 0.132, ω_1= 1.000	Not Allowed	
		2	0			
M2a	-5305.348			κ= 4.417, p_0= 0.797, p_1= 0.171, p_2= 0.031, ω_0= 0.131, ω_1= 1.000, ω_2= 1.000	None	
M7	-5305.845			κ= 4.231, *p*= 0.458, q= 1.205	Not Allowed	
		2	6.37*			
M8	-5302.662			κ= 4.309, *p0*= 0.927, (*p1*= 0.073), *p*= 0.734, *q*= 2.727, ω= 1.260	109	1.492 +- 0.159
			0.308			
M8a	-5302.816			κ= 4.2934, *p0*= 0.864, (*p1*= 0.137), *p*= 0.989, *q*= 4.737, ω= 1.000	None	

[a] The likelihood indicates the fit of the data to the different models; [b] Degree of freedom for $\chi 2$ test; [c] Result of the Likelihood ratio test amont the pair models M1a-M2a, M7-M8 and M8-M8a. *Indicate statistically significant at 95% level and **indicate statistically significant at 99% level ;[d] Pi denotes the proportion of sites falling in sites class ω_1; [e] Parameters *p* and q are the shape parameters of the beta distribution which underlies M7, M8 and M8a models

Table S6- Results obtained by PARRIS for *Ggta1* gene (LRT = -2.61186e-05).

Null model (M1)			
Rate Class	1	2	
ω	0.12	1.00	Mean : 0.354
Prob.	0.732	0.268	Std.Dev : 0.390

Alternative model (M2)				
Rate Class	1	2	3	Summary
ω	0.12	1.00	4.57	Mean : 0.354
Prob.	0.732	0.268	0.000	Std.Dev : 0.390

Table S7- Codons under positive selection obtained by Hyphy algorithms for *Ggta1* gene (values significant statistical are in highlighted in bold).

**Codon**	**SLAC dN-dS**	**SLAC p-value**	**FEL dN-dS**	**FEL p-value**	**MEME ω^+**	**MEME p-value**	**FUBAR dN-dS**	**FUBAR Post. Pr.**
82	2.012	0.249	3.036	0.072	**>100**	**0.012**	0.639	0.925
92	1.349	0.389	1.786	0.378	**57.251**	**0.035**	0.617	0.850
193	0.536	0.984	0.455	0.832	**>100**	**0.030**	-0.483	0.322
240	1.605	0.966	1.393	0.825	**>100**	**0.014**	-0.605	0.521
279	1.935	0.153	1.812	0.031	**>100**	**0.047**	0.253	0.874

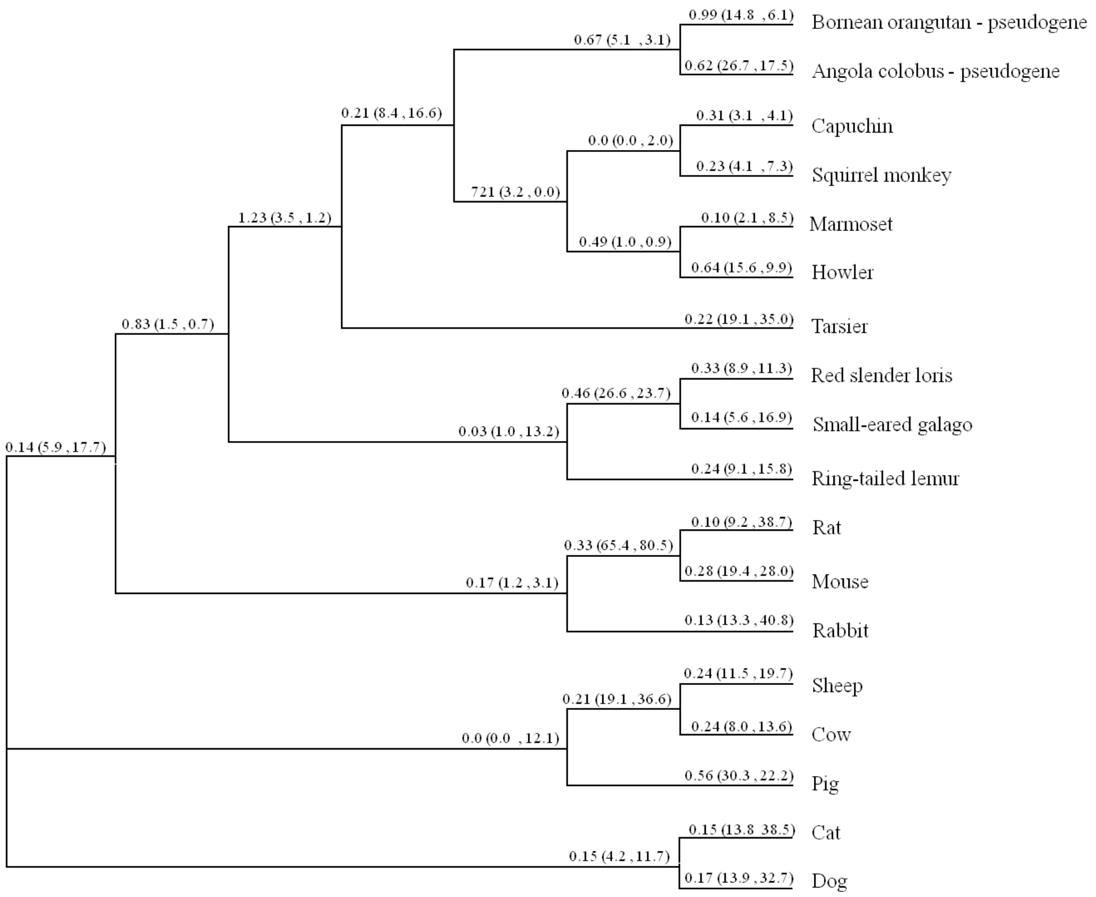

Figure S2 - Estimative of the Free-ratio model for the *Ggta1* gene. Samples with known phenotypes are indicated. The model estimated 697.3 and 286.7 nonsynonymous and synonymous sites, respectively. (M0 vs.Free-Ratio: *lnL*=- - 5363.619 and - 5332.404, respectively; LRT = 62.433; df= 33; 0.01< P< 0.05). The values on each branches corresponding to ω, nonsynonymous and synonymous sites, respectively. ω value < 0.5 indicate purifying selection, ω value > 0.5 is relaxed purifying selection and ω value > 1.0 is positive selection.

Table S8- Tests of positive selection codons in the *iGb3S* gene by pairs M0-M1a, M1a-M2a, M7-M8 and M8-M8a (PAML).

Model	lnL[a]	df[b]	LRT[c]	Estimates of Parameters[d,e]	Selected Sites	ω and SE
M0	-4244.063	1	94.85**	κ= 2.612, ω= 0.241	Not Allowed	
M1a	-4196.640			κ= 2.721, p_0= 0.728, p_1 0.272, ω_0= 0.105, ω_1= 1.000	Not Allowed	
		2	0			
M2a	-4196.640			κ= 2.721, p_0= 0.728, p_1= 0.187, p_2= 0.085, ω_0= 0.105, ω_1= 1.000, ω_2= 1.000	None	
M7	-4188.957			κ= 2.690, p= 0.364, q= 0.914	Not Allowed	
		2	5.69			
M8	-4186.112			κ= 2.688, $p0$= 0.985, ($p1$= 0.015), p= 0.427, q= 1.180, ω= 3.433	None	
			5.59**			
M8a	-4188.905			κ= 2.688, $p0$= 0.942, ($p1$= 0.058), p= 0.414, q= 1.291, ω= 1.000	Not Allowed	

[a] The likelihood indicates the fit of the data to the different models; [b] Degree of freedom for $\chi 2$ test; [c] Result of the Likelihood ratio test amont the pair models M1a-M2a, M7-M8 and M8-M8a. *Indicate statistically significant at 95% level and **indicate statistically significant at 99% level ;[d] Pi denotes the proportion of sites falling in sites class ω_1; [e] Parameters p and q are the shape parameters of the beta distribution which underlies M7, M8 and M8a models

Table S9- Results obtained by PARRIS for *iGb3S* gene (LRT = -0.00025)

Null model (M1)			
Rate Class	1	2	Summary
ω	0.13	1.00	Mean 0.372
Prob.	0.718	0.282	Std.Dev: 0.394

Alternative model (M2)				
Rate Class	1	2	3	Summary
ω	0.13	1.00	4.54	Mean 0.371
Prob.	0.719	0.281	0.000	Std.Dev 0.393

Table S10- Codons under positive selection obtained by Hyphy algorithms for *iGb3S* gene (values significant statistical are in highlighted in bold).

**Codon**	**SLAC dN-dS**	**SLAC p-value**	**FEL dN-dS**	**FEL p-value**	**MEME ω^+**	**MEME p-value**	**FUBAR dN-dS**	**FUBAR Post. Pr.**
96	3.644	0.190	**2.434**	**0.032**	>**100**	**0.009**	0.218	0.878
135	1.044	0.544	1.635	0.542	>**100**	**0.035**	-0.071	0.501
228	1.654	0.383	1.112	0.220	>**100**	**0.001**	0.048	0.620
233	2.664	0.281	4.846	0.112	>**100**	**0.024**	0.567	0.853

Table S11- Tests of positive selection codons in the *GBGT1* gene by pairs M0-M1a, M1a-M2a, M7-M8 and M8-M8a (PAML).

Model	lnL[a]	df[b]	LRT[c]	Estimates of Parameters[d,e]	Selected Sites	ω and SE
M0	-4055.301	1	89.85**	κ= 4.829, ω=0.149	Not Allowed	
M1a	-4010.374			κ= 5.478, p_0= 0.839, p_1 0.161, ω_0= 0.082, ω_1= 1.000	Not Allowed	
		2	0			
M2a	-4010.374			κ= 5.478, p_0= 0.839, p_1= 0.116; p_2= 0.045, ω_0= 0.082, ω_1= 1.000, ω_2= 1.000	None	
M7	-3998.633			κ= 5.223, *p*= 0.344, q= 1.581	Not Allowed	
		2	0.15			
M8	-3998.558			κ= 2.688, *p0*= 0.978, (*p1*= 0.021), *p*= 0.374, *q*= 1.926, ω= 3.433	None	
			0.006			
M8a	-3998.561			κ= 5.223, *p0*= 0.975, (*p1*= 0.025), *p*= 0.375, *q*= 1.956, ω= 1.000	Not Allowed	

[a] The likelihood indicates the fit of the data to the different models; [b] Degree of freedom for χ2 test; [c] Result of the Likelihood ratio test amont the pair models M1a-M2a, M7-M8 and M8-M8a. *Indicate statistically significant at 95% level and **indicate statistically significant at 99% level ;[d] Pi denotes the proportion of sites falling in sites class ω_1; [e] Parameters *p* and q are the shape parameters of the beta distribution which underlies M7, M8 and M8a models

Table S12- Results obtained by PARRIS for *GBGT1* gene (LRT = 0.0001)

Null model (M1)			
Rate Class	1	2	Mean
ω	0.11	1.00	0.270
Prob.	0.821	0.179	Std.Dev: 0.341

Alternative model (M2)				
Rate Class	1	2	3	Summary
ω	0.11	1.00	1.00	Mean 0.271
Prob.	0.821	0.179	0.000	Std.Dev 0.341

Table S13- Codons under positive selection obtained by Hyphy algorithms for *GBGT1* gene (values significant statistical are in highlighted in bold).

**Codon**	**SLAC dN-dS**	**SLAC p-value**	**FEL dN-dS**	**FEL p-value**	**MEME ω^+**	**MEME p-value**	**FUBAR dN-dS**	**FUBAR Post. Pr.**
18	-0.672	0.893	-0.198	0.771	**>100**	**0.016**	-0.077	0.262
21	2.061	0.599	1.241	0.405	**>100**	**0.018**	-0.010	0.552
237	-1.925	0.902	-0.885	0.565	**13.072**	**0.046**	-0.226	0.213
266	-2.625	0.958	-1.361	0.272	**29.819**	**0.025**	-0.247	0.139

Table S14- Tests of positive selection codons in the *GT6m7* pseudogene by pairs by pair M0-M1a, M1a-M2a, M7-M8 and M8-M8a (PAML).

Model	lnL[a]	df[b]	LRT[c]	Estimates of Parameters[d,e]	Selected Sites	ω and SE
M0	-4347.778	1	105.09**	κ= 3.096 , ω= 0.239	Not Allowed	
M1a	-4295.233			κ= 3.647, p_0= 0.637, p_1 0.363, ω_0= 0.139, ω_1= 1.000	Not Allowed	
		2	0			
M2a	-4295.233			κ= 3.647, p_0= 0.637, p_1= 0.309, p_2= 0.053, ω_0= 0.139, ω_1= 1.000, ω_2= 1.000	None	
M7	-4262.267			κ= 3.298, *p*= 0.612, q= 1.560	Not Allowed	
		2	0.002			
M8	-4262.268			κ= 3.298, *p0*= 0.999, (*p1*= 0.00001), *p*= 0.612, *q*= 1.560, ω= 1.000	None	
			0.18			
M8a	-4262.178			κ= 3.296, *p0*= 0.999, (*p1*= 0.00001), *p*= 0.609, *q*= 1.558, ω= 1.000	Not Allowed	

[a] The likelihood indicates the fit of the data to the different models; [b] Degree of freedom for χ2 test; [c] Result of the Likelihood ratio test among the pair models M1a-M2a, M7-M8 and M8-M8a. *Indicate statistically significant at 95% level and **indicate statistically significant at 99% level ;[d] Pi denotes the proportion of sites falling in sites class ω_1; [e] Parameters *p* and q are the shape parameters of the beta distribution which underlies M7, M8 and M8a models

.

Table S15- Results obtained by PARRIS for *GT6m7* pseudogene (LRT = -0.0021)

Null model (M1)			
Rate Class	1	2	Summary
ω	0.13	1.00	Mean 0.475
Prob.	0.603	0.397	Std.Dev 0.427

Alternative model (M2)				
Rate Class	1	2	3	Summary
ω	0.13	1.00	4.42	Mean 0.475
Prob.	0.602	0.398	0.000	Std.Dev 0.427

Table S16- Codons under positive selection obtained by Hyphy algorithms for *GT6m7* pseudogene (values significant statistical are in highlighted in bold).

Codon	SLAC dN-dS	SLAC p-value	FEL dN-dS	FEL p-value	MEME ω^+	MEME p-value	FUBAR dN-dS	FUBAR Post. Pr.
5	-0.191	0.845	-0.093	0.736	**>100**	**0.002**	-0.054	0.304
6	0.494	0.345	0.516	0.254	**>100**	**0.001**	-0.022	0.623
12	0.648	0.208	**0.492**	**0.052**	**>100**	**0.071**	0.135	0.842
42	-0.018	0.698	-0.057	0.916	**>100**	**0.009**	-0.066	0.334
88	0.799	0.125	0.639	0.039	**>100**	**0.046**	0.155	0.857
**96**	**1.277**	**0.048**	**1.149**	**0.017**	**>100**	**0.021**	0.495	**0.960**
105	-0.374	0.900	-0.310	0.350	**>100**	**0.002**	-0.121	0.179
107	0.066	0.610	-0.047	0.941	**>100**	**0.034**	-0.025	0.468

Figure S3- Alignment obtained by BLASTN Ensembl of intron 2 of the GT6M7 (query sequence) against Human GRCh38. Are shown the four best hits. (A) Intron2 against ADGRL3-AS1; (B)Intron2 against RUNX1 (C) Intron2 against SRBD1; (D) Intron2 against CTNND2 and (E) Intron2 against FEZ2. The exon is highlighted in red FEZ2.

A)

BLAST/BLAT type: BLASTN

Query location:1 768 to 1117 (+)

Database locatin: 4 62101554 to 62101904 (+)

Genomic location: 4 62101554 to 62101904 (+)

Alignment score: 490

E-value: 1e-133

Alignment length: 351

Percentage identity: 92.88

```
Query    Query_1:     768 TATGGTTTGGCTCTTTCCCCACCCAAATCTCAACTTGAATTGTATCTCCCAGAATTCCCA Query_1:   827
                          |||||||||||| | |||||||||||||||||||||||| ||||||||||||||||||||
Subject        4:62101554 TATGGTTTGGCTGTGTCCCCACCCAAATCTCAACTTGAACTGTATCTCCCAGAATTCCCA 4:62101613


Query    Query_1:     828 CGTGTTGTGGGAGGGACCC-GGGGGAGGTAATTGAATCATGGGGGCTGGTCTTTCCCATG Query_1:   887
                           |||||||||||||||||| |||||||||||||||||||||||| |||||||||||||||
Subject        4:62101614 TGTGTTGTGGGAGGGACCCAGGGGGAGGTAATTGAATCATGGGGACTGGTCTTTCCCATG 4:62101673


Query    Query_1:     888 CTATTCTCATGATAGTGAATACGTCTCATGAGATCTGATGGGTTTATCAGGGGTTTCCAC Query_1:  947
                          ||||||||||||||||||||| |||||||||||||||||||||||||||| ||||||| |
Subject        4:62101674 CTATTCTCATGATAGTGAATAAGTCTCATGAGATCTGATGGGTTTATCAGAGGTTTCCGC 4:62101733


Query    Query_1:     948 TTTTCCGTCTTTCTCATTTTCTCTTGCCGCTGCCATGGAAGACATGCCTTTTGCCTCCTG Query_1:  1007
                          |||| | |||| ||||||||||||||||||  ||||| ||||  |||||||| |||||||
Subject        4:62101734 TTTTGCTTCTTCCTCATTTTCTCTTGCCGCCACCATGTAAGAAGTGCCTTTTACCTCCTG  4:62101793


Query    Query_1:    1008 CCATGATTCTGAGGCCTCCCCAGCCATGTGGAACTGTAAGTCCAATTAAACCTCTTTTTC Query_1:  1067
                          |||||||| |||| ||||||||||||||||||||||||||||||||||||   |||||||
Subject        4:62101794 CCATGATTTTGAGACCTCCCCAGCCATGTGGAACTGTAAGTCCAATTAAAAAACTTTTTC  4:62101853


Query    Query_1:    1068 TTCCCAGTCTTGGGTATGTCTTTATCAGCAGCGTGAAAACAGACTAATACA  Query_1:  1118
                          |||||||||| ||||||||||||||||||||| ||||||||||||||||||
Subject        4:62101854 TTCCCAGTCTCGGGTATGTCTTTATCAGCAGCATGAAAACAGACTAATACA  4:62101905
```

B)

BLAST/BLAT type: BLASTN

Query location: 1 768 to 1117 (+)

Database location: 21 35804644 to 35804995 (-)

Genomic location: 21 35804644 to 35804995 (-)

Alignment score: 468

E-value: 4e-127

Alignment length: 352

Percentage identity: 92.33

```
Query    Query_1:     768 TATGGTTTGGCTCTTTCCCCACCCAAATCTCAACTTGAATTGTATCTCCCAGAATTCCCA Query_1:  827
                          |||||||||||| | |||||||||||||||||||||||||| ||||||||||||||| | 
Subject       21:35804995 TATGGTTTGGCTGTGTCCCCACCCAAATCTCAACTTGAATTATATCTCCCAGAATTCTCT  21:35804936

Query    Query_1:     828 CGTGTTGTGGGAGGGACCC-GGGGGAGGTAATTGAATCATGGGGGCTGGTCTTTCCCATG Query_1: 887
                          | ||||||||||||||||| ||||||||||||||||||||||||||||||||||||  ||
Subject       21:35804935 CATGTTGTGGGAGGGACCCAGGGGGAGGTAATTGAATCATGGGGGCTGGTCTTTCCTGTG  21:35804876

Query    Query_1:     888 CTATTCTCATGATAGTGAATACGTCTCATGAGATCTGATGGGTTTATCAGGGGTTTCCAC Query_1:  947
                          |||||||  |||||||||||| |||||| ||||||||||||||||||||||||||||| |
Subject       21:35804875 CTATTCTTGTGATAGTGAATAAGTCTCAAGAGATCTGATGGGTTTATCAGGGGTTTCCGC  21:35804816

Query    Query_1:     948 TTTTCCGTCTTTCTCATTTTCTCTTGCCGCTGCCATGG-AAGACATGCCTTTTGCCTCCT Query_1: 1007
                          |||| | |||| ||||||||||||||| |||||||||| ||||  |||||||||||||||
Subject       21:35804815 TTTTGCTTCTTCCTCATTTTCTCTTGCTGCTGCCATGGTAAGAACTGCCTTTTGCCTCCT  21:35804756

Query    Query_1:    1008 GCCATGATTCTGAGGCCTCCCCAGCCATGTGGAACTGTAAGTCCAATTAAACCTCTTTTT Query_1:  1067
                           |||||||||||||| ||| ||||||||||||||||||||||||||||||||||||||||
Subject       21:35804755 ACCATGATTCTGAGGGCTCTCCAGCCATGTGGAACTGTAAGTCCAATTAAACCTCTTTTT  21:35804696

Query    Query_1:    1068 CTTCCCAGTCTTGGGTATGTCTTTATCAGCAGCGTGAAAACAGACTAATACA  Query_1:  1119
                          |||||||||||  ||||||||||||||||||| |||||||||||||||||||
Subject       21:35804695 CTTCCCAGTCTCAGGTATGTCTTTATCAGCAGTGTGAAAACAGACTAATACA  21:35804642
```

C)

BLAST/BLAT type: BLASTN

Query location: 1 768 to 1117 (+)

Database location: 2 45591918 to 45592268 (+)

Genomic location: 2 45591918 to 45592268 (+)

Alignment score: 466

E-value: 1e-126

Alignment length: 351

Percentage identity: 92.02

```
Query    Query_1:      768 TATGGTTTGGCTCTTTCCCCACCCAAATCTCAACTTGAATTGTATCTCCCAGAATTCCCA Query_1:  827
                           |||||||||||| | | |||||||||||||||||||||||||||||||||||||||||||
Subject        2:45591918 TATGGTTTGGCTATGTGCCCACCCAAATCTCAACTTGAATTGTATCTCCCAGAATTCCCA  2:45591977


Query    Query_1:      828 CGTGTTGTGGGAGGGACCC-GGGGGAGGTAATTGAATCATGGGGGCTGGTCTTTCCCATG Query_1: 887
                           |||||||| |||||||||| ||||||||||||||||||||||||||  |||| |||  ||
Subject        2:45591978 CGTGTTGTAGGAGGGACCCAGGGGGAGGTAATTGAATCATGGGGGCCAGTCTCTCCTGTG  2:45592037


Query    Query_1:      888 CTATTCTCATGATAGTGAATACGTCTCATGAGATCTGATGGGTTTATCAGGGGTTTCCAC Query_1: 947
                           |||||||   ||||||||||| ||||||  ||||||||||||||||||||||||||||||
Subject        2:45592038 CTATTCTTGCGATAGTGAATAAGTCTCACCAGATCTGATGGGTTTATCAGGGGTTTCCAC  2:45592097


Query    Query_1:      948 TTTTCCGTCTTTCTCATTTTCTCTTGCCGCTGCCATGGAAGACATGCCTTTTGCCTCCTG Query_1:  1007
                           |||| | |||| ||||||||||||||| ||||||||| ||||  ||||||||||||||
Subject        2:45592098 TTTTGCTTCTTCCTCATTTTCTCTTGCTGCTGCCATGTAAGAAGTGCCTTTTGCCTCCCA  2:45592157


Query    Query_1:     1008 CCATGATTCTGAGGCCTCCCCAGCCATGTGGAACTGTAAGTCCAATTAAACCTCTTTTTC Query_1: 1067
                           |||||||||||||||||||||||||||||||||||||||||||||||||||| |||||||
Subject        2:45592158 CCATGATTCTGAGGCCTCCCCAGCCATGTGGAACTGTAAGTCCAATTAAACCACTTTTTC  2:45592217


Query    Query_1:     1068 TTCCCAGTCTTGGGTATGTCTTTATCAGCAGCGTGAAAACAGACTAATACA  Query_1:  1118
                           ||||||||||||||||||||||| |||||||| ||||||||||||||||||
Subject        2:45592218 TTCCCAGTCTTGGGTATGTCTTTGTCAGCAGCATGAAAACAGACTAATACA   2:45592269
```

D)

BLAST/BLAT type: BLASTN

Query location: 1 768 to 1117 (+)

Database location: 5 11312296 to 11312643 (-)

Genomic location: 5 11312296 to 11312643 (-)

Alignment score: 462

E-value: 2e-125

Alignment length: 351

Percentage identity: 92.02

```
Query    Query_1:     768 TATGGTTTGGCTCTTTCCCCACCCAAATCTCAACTTGAATTGTATCTCCCAGAATTCCCA Query_1: 827
                          |||||||||||| | ||||||||||||||||| |||||||||||||||||||||||||||
Subject        5:11312643 TATGGTTTGGCTGTGTCCCCACCCAAATCTCAGCTTGAATTGTATCTCCCAGAATTCCCA  5:11312584

Query    Query_1:     828 CGTGTTGTGGGAGGGACCC-GGGGGAGGTAATTGAATCATGGGGGCTGGTCTTTCCCATG Query_1:  887
                          | ||||||| ||||||||| |||| ||||||||||||||||||||| ||||||||||||
Subject        5:11312583 CATGTTGTGAGAGGGACCCAGGGGAAGGTAATTGAATCATGGGGGCCGGTCTTTCCCATC  5:11312524

Query    Query_1:     888 CTATTCTCATGATAGTGAATACGTCTCATGAGATCTGATGGGTTTATCAGGGGTTTCCAC Query_1:  947
                          |||||||| |||||||||||| |||||||||||||| ||||| ||||||||||||||  |
Subject        5:11312523 CTATTCTCGTGATAGTGAATAAGTCTCATGAGATCTAATGGGCTTATCAGGGGTTTCTGC  5:11312464

Query    Query_1:     948 TTTTCCGTCTTTCTCATTTTCTCTTGCCGCTGCCATGGAAGACATGCCTTTTGCCTCCTG Query_1:  1007
                          |||| | |||   |||||||||||||||||||||||| ||||  ||||||| |||||| |
Subject        5:11312463 TTTTGCTTCT---TCATTTTCTCTTGCCGCTGCCATGTAAGAACTGCCTTTCGCCTCCCG  5:11312404

Query    Query_1:    1008 CCATGATTCTGAGGCCTCCCCAGCCATGTGGAACTGTAAGTCCAATTAAACCTCTTTTTC Query_1:  1067
                          ||||||||||||||||||||||||||||||||||||||||||| |||||||| |||||||
Subject        5:11312403 CCATGATTCTGAGGCCTCCCCAGCCATGTGGAACTGTAAGTCCGATTAAACCCCTTTTTC  5:11312344

Query    Query_1:    1068 TTCCCAGTCTTGGGTATGTCTTTATCAGCAGCGTGAAAACAGACTAATACA          Query_1:  1118
                          |||||||||||||||||||||||||||||||| ||||||||||||||||||
Subject        5:11312343 TTCCCAGTCTTGGGTATGTCTTTATCAGCAGCATGAAAACAGACTAATACA  5:11312295
```

E)

BLAST/BLAT type: BLASTN

Query location: 1 4 to 338 (+)

Database location: 2 36594350 to 36594685 (-)

Genomic location: 2 36594350 to 36594685 (-)

Alignment score: 460

E-value: 1e-125

Alignment length: 336

Percentage identity:

```
Query    Query_1:       4 GGTTTGGCTCTTTCCCCACCCAAATCTCAACTTGAATTGTATCTCCCAGAATTCCCACGT Query_1:  63
                          ||||||||| | ||||||||||||||||||||||||||||||||||||||||||||||||
Subject        2:36594685 GGTTTGGCTGTGTCCCCACCCAAATCTCAACTTGAATTGTATCTCCCAGAATTCCCACGT  2:36594626

Query    Query_1:      64 GTTGTGGGAGGGA-CCCGGGGGAGGTAATTGAATCATGGGGGCTGGTCTTTCCCATGCTA Query_1:  123
                          ||||||||||||| || |||||||||||||||||||||||||||||||||||| ||||||
Subject        2:36594625 GTTGTGGGAGGGATCCAGGGGGAGGTAATTGAATCATGGGGGCTGGTCTTTCCTATGCTA  2:36594566

Query    Query_1:     124 TTCTCATGATAGTGAATACGTCTCATGAGATCTGATGGGTTTATCAGGGGTTTCCACTTT Query_1:  183
                          |||||||||||||||||| |||| |  |||||||||||| ||||||||||||||| ||||
Subject        2:36594565 TTCTCATGATAGTGAATAAGTCTTACAAGATCTGATGGGCTTATCAGGGGTTTCCGCTTT  2:36594506

Query    Query_1:     184 TCCGTCTTTCTCATTTTCTCTTGCCGCTGCCATGGAAGACATGCCTTTTGCCTCCTGCCA Query_1: 243
                          | |  ||| ||||||||| ||||| || |||||| ||||  |||||||||||||||||||
Subject        2:36594505 TGCTCCTTCCTCATTTTCCCTTGCTGCCGCCATGTAAGAAGTGCCTTTTGCCTCCTGCCA  2:36594446

Query    Query_1:     244 TGATTCTGAGGCCTCCCCAGCCATGTGGAACTGTAAGTCCAATTAAACCTCTTTTTCTTC Query_1:  303
                          ||||||||||||||||||||||||||||||||||||||||||||||||||| ||||||||
Subject        2:36594445 TGATTCTGAGGCCTCCCCAGCCATGTGGAACTGTAAGTCCAATTAAACCTCGTTTTCTTC  2:36594386

Query    Query_1:     304 CCAGTCTTGGGTATGTCTTTATCAGCAGCGTGAAAA  Query_1:  339
                          ||||||| |||||||||||||||||| || ||||||
Subject        2:36594385 CCAGTCTCGGGTATGTCTTTATCAGCTGCATGAAAA  2:36594349
```
